# Supplementary material for: In Vitro Digestion and Intestinal Absorption of Mycotoxins Due to Exposure from Breakfast Cereals: Implications for Children’s Health
Source: Toxins (Basel). 2024 Apr 25;16(5):205. doi: 10.3390/toxins16050205 (PMC11126104; doi:10.3390/toxins16050205)
Supplement: Supplementary file 1 [file toxins-16-00205-s001.zip › toxins-2956127-supplementary.pdf]

Supplementary Materials: In Vitro Digestion and Intestinal Absorption of Mycotoxins Due to Exposure from Breakfast Cereals: Implications for Children's Health

Table S1. Regression equations, coefficients of correlation (r) for the mycotoxins under study in bioaccessible fractions.

	**Mycotoxin**	**Matrix**	***m***	***b***	***r***	**LOD (µg/L)**	**LOQ (µg/L)**
**Gastric**	**AFB1**	C	-	-	-	-	-
		CSSM	-	-	-	-	-
		CSSMLF	-	-	-	-	-
		CSB	-	-	-	-	-
	**ENNB**	C	0.4169	6.5665	0.9975	0.4	1.2
		CSSM	0.3291	3.6091	0.9977	0.3	1.1
		CSSMLF	0.8963	-15.196	0.9910	0.7	2.3
		CSB	0.2494	7.3852	0.9834	0.9	3.1
	**STG**	C	-	-	-	-	-
		CSSM	-	-	-	-	-
		CSSMLF	-	-	-	-	-
		CSB	-	-	-	-	-
**Intestinal**	**AFB1**	C	0.0073	-0.2087	0.9908	0.7	2.3
		CSSM	0.0035	-0.0872	0.9889	0.8	2.5
		CSSMLF	0.0035	-0.0717	0.9940	0.6	1.9
		CSB	0.0049	-0.0147	0.9992	0.2	0.6
	**ENNB**	C	0.3690	0.2254	0.9987	0.3	0.8
		CSSM	0.4543	0.9663	0.9997	0.1	0.4
		CSSMLF	0.3797	11.611	0.9989	0.2	0.8
		CSB	0.3284	7.6747	0.9930	0.6	2.0
	**STG**	C	0.0709	-1.491	0.9936	0.6	1.9
		CSSM	0.0257	-0.0365	0.9975	0.4	1.2
		CSSMLF	0.0495	-0.8554	0.9961	0.4	1.5
		CSB	0.0302	0.338	0.9976	0.3	1.2

Abbreviations: C – Cereal; CSSM – Cereal with Semi-Skimmed Milk; CSSMLF – Cereal with Semi-Skimmed Milk Lactose Free; CSB – Cereal with Soy Beverage.

Table S2. Recoveries (%), repeatability and reproducibility (%CV), limits of detection (LOD) and quantification (LOQ) of the analytical method used to determine the mycotoxins under study in the bioaccessible fractions (gastric and intestinal) after *in vitro* digestion.

		Bioaccessible fractions										
		Gastric						Intestinal				
**Matrix**	**Mycotoxin**	**Spiked level (µg/L)**	**Recovery (%)**	**Repeatability (%CV)**	**Reproducibility (%CV)**	**LOD (µg/L)**	**LOQ (µg/L)**	**Recovery (%)**	**Repeatability (%CV)**	**Reproducibility (%CV)**	**LOD (µg/L)**	**LOQ (µg/L)**
**C**	**AFB1**	25	-	-	-	-	-	102.6	6.78	3.0	0.2	0.8
		50	-	-	-	-	-	81.4	10.46	14.7		
		200	-	-	-	-	-	74.1	8.31	6.8		
	**ENNB**	25	97.4	5.73	14.6	0.5	1.8	61.2	5.13	1.0	0.5	1.7
		50	90.2	6.09	6.7			82.5	9.57	14.4		
		200	72.4	3.44	5.3			84.4	9.91	4.4		
	**STG**	25	-	-	-	-	-	80.3	5.94	7.3	0.6	1.9
		50	-	-	-	-	-	101.6	4.8	8.3		
		200	-	-	-	-	-	115.3	11.51	1.2		
**CSSM**	**AFB1**	25	-	-	-	-	-	117.9	9.33	0.4	0.5	1.8
		50	-	-	-	-	-	94.4	12.97	9.2		
		200	-	-	-	-	-	93.1	5.86	1.5		
	**ENNB**	25	94.0	6.65	3.2	0.6	2.0	102.9	8.8	1.0	0.3	1.0
		50	83.4	11.0	3.8			89.9	5.51	7.6		
		200	67.8	7.5	7.2			58.2	8.1	0.2		
	**STG**	25	-	-	-	-	-	104.3	7.99	4.5	0.2	0.8
		50	-	-	-	-	-	94.6	9.6	12.4		
		200	-	-	-	-	-	71.3	8.0	10.2		
**CSSMLF**	**AFB1**	25	-	-	-	-	-	113.2	7.9	3.4	0.1	0.4
		50	-	-	-	-	-	93.2	5.81	1.0		
		200	-	-	-	-	-	99.4	12.0	0.9		
	**ENNB**	25	105.6	8.64	2.5	0.6	2.1	110.3	8.31	10.2	0.1	0.4
		50	117.8	6.36	0.5			91.1	7.9	15.3		
		200	97.0	7.51	13.2			72.2	9.84	8.5		
	**STG**	25	-	-	-	-	-	55.0	6.1	0.1	0.5	1.8
		50	-	-	-	-	-	63.8	9.68	0.8		
		200	-	-	-	-	-	68.2	14.2	11.7		
**CSB**	**AFB1**	25	-	-	-	-	-	117.9	8.6	0.9	0.3	1.0
		50	-	-	-	-	-	87.2	3.53	11.4		
		200	-	-	-	-	-	55.5	7.68	8.1		
	**ENNB**	25	100.1	10.9	3.0	0.4	1.3	99.5	10.5	11.5	0.7	2.4
		50	97.2	8.0	5.3			110.1	6.75	12.8		
		200	81.0	9.7	4.2			56.2	6.91	3.8		
	**STG**	25	-	-	-	-	-	108.7	6.61	12.1	0.5	1.6
		50	-	-	-	-	-	119.1	3.6	0.3		
		200	-	-	-	-	-	98.7	13.7	4.7		

Table S3. Regression equations, coefficients of correlation (r) for the mycotoxins under study in transport assay.

Mycotoxin	Matrix	*m*	*b*	*r*	LOD (µg/L)	LOQ (µg/L)
**AFB1**	C	0.0019	-0.0222	0.9899	0.4	1.2
	CSSM	0.0025	-0.0079	0.9966	0.2	0.7
**ENNB**	C	0.224	0.3777	0.9976	0.2	0.6
	CSSM	0.1751	0.4629	0.9997	0.1	0.2
**STG**	C	0.0227	-0.3864	0.9842	0.5	1.5
	CSSM	0.0021	-0.0164	0.9951	0.2	0.8
